# Coexpression Clusters and Allele-Specific Expression in Metabolism-Based Herbicide Resistance

**DOI:** 10.1093/gbe/evaa191

**Published:** 2020-09-11

**Authors:** Darci A Giacomini, Eric L Patterson, Anita Küpper, Roland Beffa, Todd A Gaines, Patrick J Tranel

**Affiliations:** 1 Department of Crop Sciences, University of Illinois Urbana-Champaign; 2 Department of Plant, Soil and Microbial Sciences, Michigan State University; 3 Bayer AG, Division of Crop Science, Frankfurt, Germany; 4 Department of Agricultural Biology, Colorado State University

**Keywords:** nontarget-site resistance (NTSR), *Amaranthus tuberculatus*, differential expression analysis, single-nucleotide polymorphism (SNP) analysis, 4-hydroxyphenylpyruvate dioxygenase (HPPD), 2,4-dichlorophenoxyacetic acid (2,4-D)

## Abstract

In the last decade, *Amaranthus tuberculatus* has evolved resistance to 2,4-dichlorophenoxyacetic acid (2,4-D) and 4-hydroxyphenylpyruvate dioxygenase inhibitors in multiple states across the midwestern United States. Two populations resistant to both mode-of-action groups, one from Nebraska (NEB) and one from Illinois (CHR), were studied using an RNA-seq approach on F_2_ mapping populations to identify the genes responsible for resistance. Using both an *A. tuberculatus* transcriptome assembly and a high-quality grain amaranth (*A. hypochondriacus*) genome as references, differential transcript and gene expression analyses were conducted to identify genes that were significantly over- or underexpressed in resistant plants. When these differentially expressed genes (DEGs) were mapped on the *A. hypochondriacus* genome, physical clustering of the DEGs was apparent along several of the 16 *A. hypochondriacus* scaffolds. Furthermore, single-nucleotide polymorphism calling to look for resistant-specific (R) variants, and subsequent mapping of these variants, also found similar patterns of clustering. Specifically, regions biased toward R alleles overlapped with the DEG clusters. Within one of these clusters, allele-specific expression of *cytochrome*  *P450*  *81E8* was observed for 2,4-D resistance in both the CHR and NEB populations, and phylogenetic analysis indicated a common evolutionary origin of this R allele in the two populations.

**Significance**

The evolution of nontarget-site herbicide resistance is a challenge for weed management and is poorly understood. Mapping of genes differentially expressed between herbicide-sensitive and -resistant *Amaranthus tuberculatus* plants revealed physical clustering of some of them in the genome. We also identify a gene encoding a cytochrome P450 that potentially contributes to resistance to 2,4-D and is evolutionarily related in two geographically distant populations.

## Introduction

If left uncontrolled, weeds can decrease the yields of several major crops by more than 50% in present North American agronomic systems (Soltani et al. 2016, 2018). Many growers in the United States currently rely heavily on chemical means (i.e., herbicides) to control their weed populations, but the effectiveness of this approach is steadily declining due to growing numbers of herbicide-resistant weeds. Although herbicide resistance has been present in the United States since the late 1950s (Hilton 1957; Switzer 1957), the widespread adoption of herbicide-tolerant crop varieties in the mid-1990s and overreliance on one or two herbicidal modes of action contributed to an exponential increase in the number of resistant weed species over the last two decades (Heap 2020). There are currently 164 weed species in the United States with documented resistance to herbicides spanning 1 or more modes of action (Heap 2020).

From a practical standpoint, understanding how weeds deal with herbicidal compounds to avoid damage is a major goal of weed science, both to generate workarounds to combat herbicide resistance and to gain insights into plant evolution. Research on herbicide-resistance mechanisms over the last several decades has largely been focused on mutations occurring within genes that encode the target enzymes that are directly inhibited by herbicides (target-site resistance). Only recently has significant progress been made on nontarget-site-based resistance (NTSR) mechanisms, largely due to the increased availability of high-throughput whole genome/transcriptome analyses (Gaines et al. 2014; Duhoux et al. 2015; Bai et al. 2018; Gil et al. 2018). This work has largely pointed to enhanced herbicide metabolism as a primary route of NTSR, but resistance mechanisms including reduced translocation (Goggin et al. 2016) and vacuolar sequestration (Ge et al. 2010) have also been reported.

From an academic standpoint, widespread use of herbicides to control weeds provides an excellent platform for studying rapid adaptation of plants to strong selection, and to address evolutionary questions that are increasingly tractable due to genomics advances. For example, the relative contributions of new mutations, standing genetic variation, and gene flow to convergent evolution of herbicide resistance at a landscape scale recently was addressed using genomics and population-genetics approaches in the agriculturally important weed *A. tuberculatus* (Kreiner et al. 2019).

*Amaranthus tuberculatus* is a highly problematic weed species for growers across the midwestern United States, due to both its high fecundity and ability to readily evolve resistance to herbicides. Since the report of acetolactate synthase (ALS)-inhibitor resistance in *A. tuberculatus* in 1993 (Horak and Peterson 1995), this species has accrued resistances to herbicides spanning six additional sites of action (Heap 2020). In 2016, a population was discovered in Illinois that carried five-way resistance, including resistance to photosystem II inhibitors, protoporphyrinogen oxidase (PPO) inhibitors, 4-hydroxyphenylpyruvate dioxygenase (HPPD) inhibitors, and synthetic auxins (Evans et al. 2019). Two of the resistance traits (ALS and PPO) were found to be attributable to target-site mutations, but both the HPPD-inhibitor- and synthetic auxin-resistance mechanisms were unknown. In 2012, a population was reported from Nebraska that was highly resistant to 2,4-D (Bernards et al. 2012) and was subsequently determined to be resistant to HPPD-inhibiting herbicides as well (Murphy and Tranel 2019).

As is the case with other weeds, genes responsible for NTSR in *A. tuberculatus* are largely unknown. Enhanced herbicide metabolism via glutathione *S*-transferases (GSTs) and cytochrome P450 monooxygenases (CYP450s) has been described in both HPPD-inhibitor resistance (Ma et al. 2013; Kaundun et al. 2017; Shergill et al. 2018; Kohlhase et al. 2019) as well as for 2,4-D resistance (Figueiredo et al. 2018). An RNA-seq study of HPPD-inhibitor-treated *A. tuberculatus* populations revealed 29 CYP450 genes that were upregulated in resistant plants compared with sensitive plants, including one (*CYP72A15*) that was induced 3 h after treatment along with ten more CYP450s upregulated 24 h after treatment (Kohlhase et al. 2019). Resistance to 2,4-D in another *A. tuberculatus* population was also attributed to enhanced 2,4-D metabolism possibly mediated by a cytochrome P450 (Figueiredo et al. 2018). Similarly, ALS-inhibitor resistance likely can be conferred by constitutive or upregulated expression of specific CYP450s (Shergill et al. 2018) and herbicide metabolism genes have been implicated in PPO-inhibitor resistance (Obenland et al. 2019) in *A. tuberculatus*. For PS-II inhibitors, a phi-class GST (*AtuGSTF2*) has been identified and is known to confer resistance to atrazine (Evans et al. 2017). Clearly, enhanced metabolism of herbicides is an important NTSR mechanism in *A. tuberculatus*, however, in almost all studies published so far, the specific gene(s) responsible has not been identified.

In this paper, we present the results of an RNA-seq study conducted on the Illinois and Nebraska *A. tuberculatus* populations with resistance to both HPPD inhibitors and synthetic auxins. The objectives of this research were to: 1) use RNA-Seq on F_2_ mapping populations to measure differential gene expression between resistant and sensitive F_2_ individuals for both populations and both herbicides, 2) explore the origin and regulation of select differentially expressed genes (DEGs), and 3) use genomics-based analysis to understand the patterns of gene expression and the role of coexpression clusters in herbicide resistance.

## Materials and Methods

### F_2_ Production and Tissue Collection

Two populations of *A. tuberculatus* showing resistance to HPPD inhibitors and 2,4-D were identified from both Illinois (referred to as “CHR”) (Evans et al. 2019) and Nebraska (referred to as “NEB”) (Bernards et al. 2012). Herbicide-resistant plants from each population were crossed with an herbicide-sensitive *A. tuberculatus* population (WUS; originally collected in Brown County, OH) and F_1_ seeds were screened to confirm resistance to both HPPD inhibitors and 2,4-D. To screen these F_1_ populations, plants were grown under previously described greenhouse conditions (Lillie et al. 2020) and sprayed with an initial discriminating dose of mesotrione (220 g ai ha^−1^; Callisto; Syngenta Crop Protection Inc., Greensboro, NC) plus 1% v/v crop oil concentrate, followed by a late POST treatment of 2,4-D (560 g ae ha^−1^; 2,4-D amine; Nufarm Americas Inc., Chicago, IL) plus 0.25% v/v nonionic surfactant. All herbicide applications were made using a moving-nozzle spray chamber as described previously (Lillie et al. 2020). Within each of the NEB- and CHR-derived F_1_ lines, pairs of full-sibling F_1_ survivors were crossed together to form several segregating pseudo-F_2_ populations. Because *A. tuberculatus* is dioecious, an F_1_ plant cannot be selfed to create a true F_2_ population.

A single pseudo-F_2_ (hereafter referred to as an F_2_) population was selected each from NEB and CHR and several hundred seeds from each F_2_ were germinated for 48 h on wet filter paper in a growth chamber set to a 12-h day/night cycle (35 °C/15 °C). Germinated seedlings were transplanted into 50-cm^3^ pots filled with Weed Lite Mix (3:1:1:1 mixture of LC1 [Sun Gro Horticulture Canada]: Soil: Peat: Torpedo Sand) and grown in the greenhouse until plants reached a height of 4 − 6 cm. One hundred plants from each F_2_ population were then transplanted into 3.8-l round pots filled with Weed Lite Mix and allowed to grow until plants reached 8 − 10 cm in height. Tissue was then collected from the smallest fully unfolded leaf, immediately placed into liquid nitrogen, and stored at −80 °C until RNA extraction. All tissue was collected within a 2-h period between 10 AM and noon on the same day. Tissue was taken prior to herbicide application and herbicide-treated tissue was not included in this study. Without the use of an extensive (and expensive) time course RNAseq study, identifying potential resistance genes that are induced by herbicide application is extremely difficult due to the differential effects of herbicide treatment on stress and death pathways between resistant and sensitive plants (Giacomini et al. 2018).

All F_2_ plants continued to grow for three more weeks until each plant had produced multiple side shoots, at which point the side shoots were clipped off, dipped in rooting hormone, and transplanted into 400-cm^3^ inserts in flats filled with damp soil. These flats were covered with a clear 15-cm plastic dome (to maintain high humidity) until the clones established a good root system (∼3 − 4 weeks). Four clones were produced from each plant and each clone was treated with either an HPPD inhibitor or 2,4-D at a high or low dose to phenotype each F_2_ individual for multiple herbicide resistance. The low and high rates of HPPD inhibitor were 27 and 270 g tembotrione ha^−1^ (Laudis; Bayer CropScience LP, Research Triangle Park, NC), respectively. The low and high rates of 2,4-D were 560 and 2,240 g ae ha^−1^ (2,4-D amine; Nufarm Americas Inc, Chicago, IL), respectively. Clones were visually rated for herbicide damage 14 and 21 DAT, using a 1 − 10 scale (a score of 10 indicated no plant damage).

**Fig. 1:**
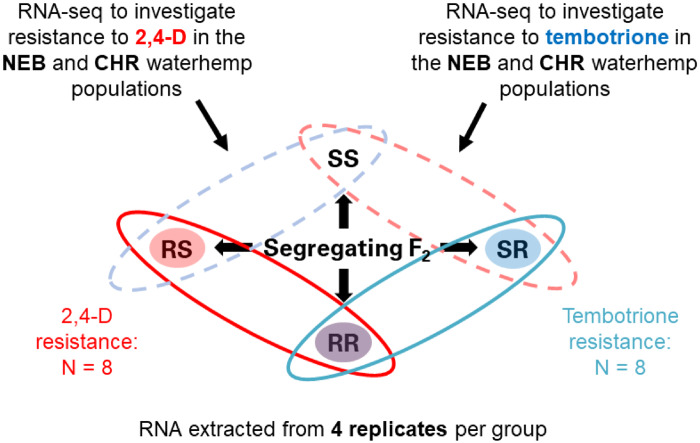
Experimental design for this study. Within each F_2_ population, plants were cloned and sprayed with high and low rates of tembotrione or 2,4-D. Based on their response, each plant was grouped into one of four categories: RR, resistant to both 2,4-D and tembotrione; RS, resistant to 2,4-D and sensitive to tembotrione; SR, sensitive to 2,4-D and resistant to tembotrione; and SS, sensitive to both 2,4-D and tembotrione. The four most resistant/sensitive plants from each category were chosen for RNA-seq analysis. This allowed for an *N *= 8 comparison between resistant and sensitive plants for each herbicide using only 16 plants for each population.

The cloning and spraying procedure was repeated on another 70 plants from each population to generate enough data for a Fisher’s exact test to assess whether the two resistance traits segregated independently of one another. Using a cutoff of 3 (Supplementary Material online; fig. 1) on the visual rating scale to score plants as either sensitive or resistant, count data for each category was fed into R and analyzed using fisher.test (alternative = “two.sided”).

### RNA Sequencing and Assembly

Based on the clonal visual ratings at both rates 21 DAT, F_2_ plants were ranked in order of least to most resistant for both tembotrione and 2,4-D. Within each F_2_ population, plants were then grouped into four categories: 1) RR, resistant to both 2,4-D and tembotrione; 2) RS, resistant to 2,4-D and sensitive to tembotrione; 3) SR, sensitive to 2,4-D and resistant to tembotrione; and 4) SS, sensitive to both 2,4-D and tembotrione. The four most resistant and sensitive in each category (16 plants total from each population and 32 plants overall) were selected for RNA extraction using a Trizol-based method (Simms et al. 1993) with a DNase I treatment following extraction. Samples were checked for quality and quantity, respectively, by running them on a Qubit analyzer and on a 1% agarose gel before sending them to the Roy J. Carver Biotechnology Center at the University of Illinois, Urbana-Champaign for Illumina library construction and sequencing.

The RNAseq libraries were prepared using the Illumina TruSeq Stranded mRNAseq Sample Prep kit (Illumina Inc., San Diego, CA, Cat. # RS-122-2101). The libraries were quantitated by qPCR and sequenced across four lanes on a HiSeq 4000 using a HiSeq 4000 sequencing kit version 1. Fastq files were generated and demultiplexed with the bcl2fastq v2.17.1.14 Conversion Software (Illumina). Adaptors were trimmed from the 3′ end of the reads and any leading or trailing bases below a quality score of 30 were trimmed via Trimmomatic-0.33, only retaining reads that were 30-bp or longer (Bolger et al. 2014).

The trimmed read files within each subgroup (RR, RS, SR, and SS) were concatenated and assembled using Trinity v2.1.0 (Grabherr et al. 2011). All four resulting assemblies were compared with one another and clustered into groups of transcripts using CD-HIT (Li and Godzik 2006). The longest transcript from each group was used as a representative of that group, generating a final reference transcriptome.

### Differential Transcript and Gene Expression Analysis

Each sample was aligned to the reference transcriptome assembly using kallisto (Bray et al. 2016) with the following parameters: –b 100 –bias –single –rf-stranded –l 255 –s 40. These pseudoalignments were then analyzed for differential expression using sleuth (Pimentel et al. 2017) with herbicide sensitivity rating (R vs S) as the condition. The sleuth analysis was carried out for all four comparisons: Tembotrione resistant versus sensitive for the NEB population, tembotrione resistant versus sensitive for the CHR population, 2,4-D resistant versus sensitive for the NEB population, and 2,4-D resistant versus sensitive for the CHR population (*n* = 8). Transcripts were further mapped to gene models from a reference genome assembly of *A. hypochondriacus* (Lightfoot et al. 2017; GenBank accession number GCA_000753965.1) to calculate the gene-level differential expression and to anchor genes to scaffolds, potentially identifying any physical clustering of DEGs. GMAP (Wu and Watanabe 2005) was used to align transcripts to the genome in a splice-aware manner (–cross-species –n 1 –min-trimmed-coverage = 0.80 –min-identity = 0.80). This gene-transcript mapping table was then fed into sleuth, which was rerun in gene mode to calculate differential gene expression between herbicide-resistant and sensitive cohorts. Genes with a Benjamini−Hochberg corrected *P*-value (Benjamini and Hochberg 1995) of 0.1 or less were considered DEGs and used in further analyses.

### Coexpression Cluster Analysis

Significant clustering of the DEGs was tested using CROC (Pignatelli et al. 2009). CROC searches for clusters using a hypergeometric test that calculates the probability of getting k number of DEGs (out of *n* total genes) present in a sliding window along each scaffold. A window size of 1 Mb and an offset size of 500 kb was used, calling significant clusters only when the adjusted *P*-value (false discovery rate [FDR]) was <0.05. A sliding window approach was used to visualize clustering along each of the 16 longest scaffolds using R v3.5.1 (R Core Team 2018). Given a window size of 500 kb and a step size of 500 kb, the number of DEGs was counted within each window and plotted using a custom R script (Supplementary Material online, “slidingWindowPlots.R”). 

Additionally, over-representation of DEGs at the whole-chromosome level was tested by totaling up the number of DEGs across each chromosome and comparing them to the expected number of DEGs on that chromosome using Fisher’s Exact test in R. Adjusted *P*-values (p.adjust, method = “bonferroni”) were calculated.

### Single-Nucleotide Polymorphism Calling

Single-nucleotide polymorphisms (SNPs) were called using the best practices outlined by GATK v3.7 (Van der Auwera et al. 2013). Cleaned reads from each RNA-seq sample were first mapped to the *A. hypochondriacus* genome using STAR v2.5.3 (Dobin et al. 2013) with the following parameters: --outSAMtype BAM SortedByCoordinate --quantMode TranscriptomeSAM GeneCounts --sjdbGTFtagExonParentTranscript Parent. Read groups were assigned and PCR duplicates were removed using Picard Tools v1.95 (The Broad Institute 2019), followed by hard clipping of sequences that extended into the intronic regions using the GATK SplitNCigarReads tool. To correct for any systemic bias in the quality of each aligned base, GATK BaseRecalibrator was run using a set of high-quality SNPs. Because no high-quality SNP data sets exist for *A. tuberculatus*, a set was created from data generated herein by first running an initial round of variant calling on the uncalibrated data using GATK’s HaplotypeCaller and GenotypeGVCFs functions, then hard filtering the SNPs using the following strict parameters: QD < 2.0; FS > 60.0; MQ < 40.0; MQRankSum < −12.5; ReadPosRankSum < −8.0. After base recalibration, variant calling was again run, this time on the calibrated data, using HaplotypeCaller (parameters: -dontUseSoftClippedBases -stand_call_conf 20.0 --variant_index_type LINEAR --variant_index_parameter 128000 -ERC GVCF) and Genotype GVCFs. SNPs were extracted from the final variant file and filtered to include only SNPs that were biallelic and that passed the following parameters: -window 35 -cluster 3 -filter QD < 2.0 -filter FS > 30.0.

Out of this final SNP data set, condition-specific SNPs were called using the case/control association analysis in PLINK v1.9 (Steiß et al. 2012; Chang et al. 2015). Due to low sample sizes for each herbicide-resistant versus sensitive comparison (*n* = 8), an adaptive Monte Carlo permutation test with 1,000 iterations was also run as part of this association analysis. SNPs that were different between R and S plants with a corrected *P*-value of 0.05 or less were called as condition-specific SNPs. As with the DEGs, a sliding window approach (Supplementary Material online, “slidingWindowPlots.R”) was used to visualize these condition-specific SNPs, using a window size of 500 kb and a step size of 500 kb.

### Quantitative PCR

Of the DEGs that emerged from the data for all four comparisons, the most likely candidates for herbicide resistance were identified based on their relative rank, fold-change expression, and gene annotation as a possible metabolic resistance gene, as supported by previous papers suggesting a herbicide-metabolism-based resistance mechanism for these populations (Figueiredo et al. 2018; Evans et al. 2019). Quantitative PCR primers were developed for each candidate gene. Primers were also created for six housekeeping genes and PCR efficiencies were calculated for all primer sets using a 5-step log-scale serial dilution of cDNA. Only primer sets that showed a PCR efficiency close to 100% (+/- 5%) were retained and used for further analyses (supplementary table 1, Supplementary Material online).

To validate the differential analysis results, a subset of F_2_ plants from both CHR- and NEB-derived populations were selected (*n* = 14), including individuals that were and were not used in the RNA-seq. RNA was extracted from all samples using the Trizol method (previously described) and RNA was converted to cDNA using a ProtoScript First Strand cDNA Synthesis Kit (NEB). Quantitative PCR was performed in triplicate on each sample for each primer set by combining 5 μl of iTaq Universal SYBR Green Supermix (Bio-Rad), 0.5 μl forward primer (10 μM), 0.5 μl reverse primer (10 μM), 3 μl of nuclease-free water, and 1 μl of cDNA. Three housekeeping genes were run on each plate for each sample to serve as endogenous controls and assays were conducted 2 − 3 times to ensure consistent results. Relative expression was calculated using the 2^–ΔΔCt^ method (Livak and Schmittgen 2001), using a sensitive parent (WUS) as the reference sample. These expression values were then regressed against the phenotypic rating values in R (stats v3.6.1) to test for a significant linear relationship for each population.

### Promoter Analysis

The 1,000 bases upstream of the transcription start site were extracted from all *A. tuberculatus* genes from a newly assembled *A. tuberculatus* genome (Kreiner et al. 2019) for transcription factor-binding site (TFBS) analysis. These “promoter” regions were searched for known plant-specific TFBSs by matching them to position weight matrices downloaded from JASPAR 2018 (Khan et al. 2018). Using the searchSeq function from TFBSTools v1.10.3 in R (min.score = “90%”), all 501 plant-specific TFBSs were matched and counted across the *A. tuberculatus* promoter regions. Each DEG set was tested for over-representation of the TFBSs using a hypergeometric test in R (phyper; lower.tail = FALSE) and correcting for multiple testing using the Benjamini-Hochberg *P*-value adjustment method (p.adjust; method = “BH”).

### Allele-Specific Expression Analysis

Given the co-occurrence of both differential gene expression and condition-specific SNPs in several regions of the genome, the hypothesis of allele-specific expression (ASE) was tested using the read count data for each condition-specific SNP to identify all heterozygous individuals (those that showed expression of each allele). Homozygous resistant and sensitive plants at each SNP site were then used to classify each SNP as R or S, then the count data of each R- or S-associated SNP in the heterozygous individuals were used to test for a significant difference in read depth between R and S SNPs using R (rstatix). SNPs and their associated adjusted *P*-values (Benjamini and Hochberg, *P* = 0.1) were plotted across the scaffold 4 cluster region using R (ggpubr).

### *Cytochrome 81E8* Phylogenetic Analysis

Both the CHR and the NEB population showed the same upregulated allele of a CYP81E8 gene for resistance to 2,4-D, raising the question of whether or not this putative resistance allele evolved independently in each population. Using a previously published (Kreiner et al. 2019) data set of whole genome sequence from *A. tuberculatus* samples from Illinois and Canada, a phylogenetic tree was constructed to examine the evolutionary relationship of *CYP81E8* from each population. Whole genome or whole transcriptome data sets were aligned to the CDS of *CYP81E8* using bowtie2 (Langmead and Salzberg 2012) (parameters: --no-unal -t -L 20). The sorted bam files were then fed into the same GATK SNP pipeline described above to generate a filtered vcf file. The SNPRelate package in R converted this vcf file to a gds file that could then be used to generate a dendogram based on relatedness (snpgdsHCluster; snpgdsCutTree, n.perm = 5000).

## Results

### Resistance Response

Dose–response data from previous work have shown about a 15-fold level of resistance to mesotrione and 9-fold resistance to 2,4-D for the CHR population compared with WUS (Evans et al. 2019). A similar level of 2,4-D resistance has been reported in the NEB population, with 10-fold resistance compared with the Nebraska 2,4-D sensitive population (Bernards et al. 2012) that was reverted to sensitivity by pretreatment with the cytochrome P450 inhibitor malathion (Figueiredo et al. 2018). For tembotrione, we saw a 43-fold resistance in the CHR population and a 15-fold resistance in the NEB population, compared with WUS (Murphy and Tranel 2019). In both CHR and NEB populations, resistance to tembotrione and 2,4-D appeared to segregate independently (*P*-value = 0.2457 and 0.1457, respectively). By selecting four F_2_ plants with each resistance combination (RR, RS, SR, and SS) we were able to achieve, for each population, eight replicate comparisons for each of two resistant traits from only 16 plants (fig. 1).

### Differential Transcript/Gene Expression

The transcriptome assembled into 57,106 transcripts for a total length of 98,112,700 bp. The 32 libraries (16 for each population) were all sequenced to a minimum of 40 million reads per sample (total reads sequenced ranged from 40,800,978 to 54,938,593 bp). Over 80% of reads aligned to the transcriptome for each sample with an average of 81.3% alignment across all libraries, resulting in approximately ∼40× coverage across the entire transcriptome.

For the CHR F_2_ population, there were 39 differentially expressed transcripts (DETs) between 2,4-D resistant and 2,4-D sensitive plants and 121 DETs between tembotrione resistant and sensitive plants (supplementary table 2, Supplementary Material online). In the NEB F_2_ population, 1,445 transcripts were found to be differentially expressed between 2,4-D resistant and sensitive plants and 115 between tembotrione resistant and sensitive plants.

One of the most significantly DETs in the CHR population for 2,4-D resistance was a cytochrome P450 (*CYP81E8*), also identified as an *isoflavone 2’-hydroxylase*. This same cytochrome P450 gene was also found to be significantly overexpressed in 2,4-D resistant plants for the NEB population, pointing to a possible shared resistance mechanism between these two populations despite their disparate geographic origins. Quantitative PCR analysis validated overexpression of *CYP81E8*, finding strong correlations between its expression and phenotypic response to 2,4-D for both populations (table 1). Other putative resistance genes underwent the same qPCR validation process, confirming higher expression of a glucosyltransferase (*UDP-glucose flavonoid 3-O-glucosyltransferase*) in NEB plants resistant to the HPPD inhibitor. An ABC transporter gene that emerged as a DET for the CHR population for tembotrione was also confirmed to correlate with resistance, not only for the HPPD inhibitor, but also for 2,4-D resistance in both populations. All genes were also examined for genomic copy number increase using a qPCR-based assay, and no evidence of gene duplication for any of these DETs was found (data not shown).

**Table 1:** Linear Regression of RT-qPCR Expression Data for Each Gene Against Phenotypic Damage Ratings for Each Population (CHR and NEB) and Each Chemistry (HPPD and 2,4-D)

Gene	HPPD		2,4-D	
	CHR	NEB	CHR	NEB
*ABCI11*	NS	NS	NS	NS
*CYP81E8*	NS	NS	0.021	0.008
*ABCC10*	NS	0.036	0.034	0.018
*UDPflav*	NS	0.047	NS	NS
*CYP97B2*	NS	NS	NS	NS
*CYP71A1*	NS	NS	NS	NS
*CYP72A219*	NS	NS	NS	NS
*BTBTOZ*	NS	NS	NS	NS

Note.—Significant *P*-values reported; NS, not significant.

Differential expression was also measured at the gene level to 1) increase the power and remove any confounding information due to minor transcript isoforms and 2) be able to later map the genes to the genome for spatial gene expression profiling. For the CHR population, 90 and 31 DEGs were obtained for the 2,4-D comparison and tembotrione comparison, respectively. Again, the NEB population gave higher numbers, with 676 DEGs found for the 2,4-D comparison and 268 DEGs found in the tembotrione comparison (supplementary table 3, Supplementary Material online).

### Coexpression Cluster Analysis

**Fig. 2:**
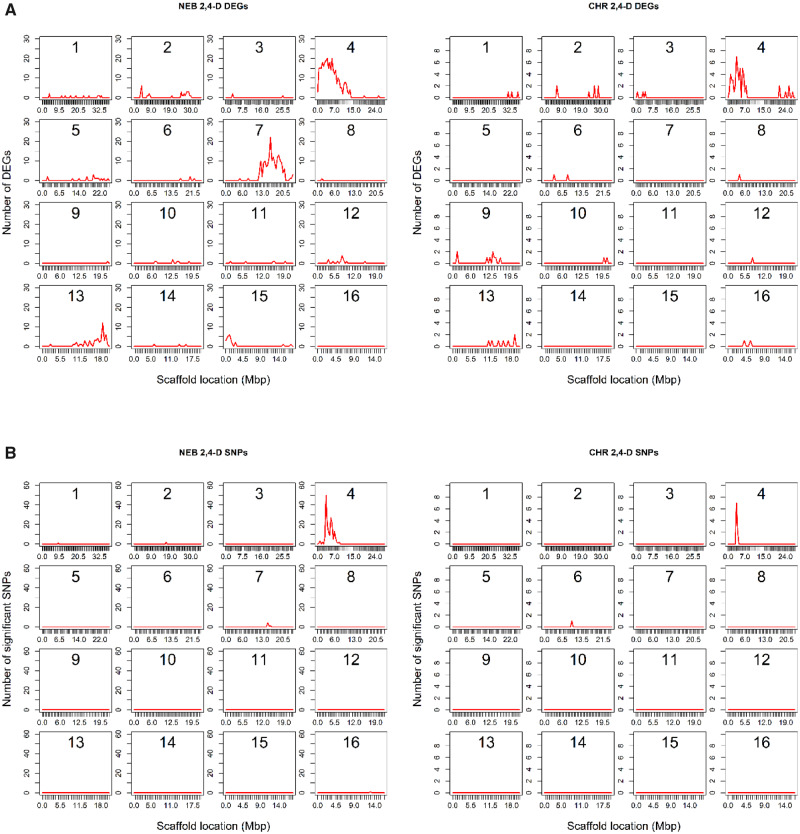
Sliding window graph of significantly differentially expressed genes and significant SNPs. (*A*) Significantly differentially expressed genes (DEGs) between 2,4-D resistant and sensitive plants in CHR and NEB mapped on the *A. hypochondriacus* genome. Only genes with an FDR of 0.05 or less were considered significant. (*B*) SNPs that were statistically different between 2,4-D resistant and sensitive plants in CHR and NEB mapped on the *A. hypochondriacus* genome. Statistically significant SNPs were called if PLINK analysis returned a corrected *P*-value of 0.05 or less.

DEGs between the 2,4-D resistant and sensitive biotypes of both CHR and NEB were found to physically cluster together in a few chromosomal regions. CROC analyses found significant clustering in a region on scaffold 4 for both populations and a significant region in scaffold 7 for the NEB population (table 2; fig. 2*A*). No significant regional clustering was observed for DEGs between HPPD-resistant and -sensitive plants, however, a Fisher’s exact test for over-representation of DEGs across the entire chromosome-level scaffolds indicated significantly higher numbers of DEGs than expected on scaffolds 6 and 13 for NEB (supplementary table 4, Supplementary Material online). This over-representation analysis also identified the significant clustering previously found for the 2,4-D comparisons on scaffold 4 (for CHR and NEB) and scaffold 7 (for NEB) as well as clustering on scaffold 13 for NEB. It may be that the low sample sizes (*n* = 8) were insufficient for adequate resolution of coexpression clusters in the HPPD comparisons.

**Table 2:** Chromosomal Cluster Testing (Using CROC; Pignatelli et al. 2009) of Differentially Expressed Genes in CHR and NEB for 2,4-D Resistance

Scaffold	Population	Start	Stop	Adj. *P*-Value
Scaffold_4	CHR	3,469,336	6,412,488	0.0058
Scaffold_4	NEB	3,002,834	9,781,978	1.37E−06
Scaffold_7	NEB	14,666,782	16,050,619	0.0021

### Condition-Specific SNPs

To check for the presence of any resistant-specific SNPs in these populations, SNPs were called across all genes and condition-specific SNPs (those that varied between resistant and sensitive plants) were identified using Fisher’s exact test in PLINK v1.9. Using an adjusted *P*-value cutoff of 0.05, 10, and 192 SNPs were found to be associated with resistance in the 2,4-D resistant versus sensitive comparison for CHR and NEB, respectively (supplementary table 5, Supplementary Material online). In both populations, SNPs were found to cluster in the same regions that DEGs were found to cluster. In CHR, 9 out of 10 SNPs were found in the region of scaffold 4 that contained the CYP81E8 gene, whereas the other SNP was found on scaffold 6. Within the scaffold 4 cluster, there were significant SNPs found in both the CYP81E8 gene as well as a PIN3 auxin efflux carrier gene (which is interesting given 2,4-D is a synthetic auxin). However, 2,4-D resistance cannot be attributed to any of these SNPs because they are in linkage disequilibrium with one another, making it challenging to locate the causal variant. Fine mapping of this region is currently underway. In NEB, 182 SNPs were found in the scaffold 4 region, 6 were found in the scaffold 7 region that also showed a cluster of DEGs in the expression analysis, and the other 4 SNPs were scattered across scaffolds 1, 2, and 16. Sliding window graphs illustrate the clustering of these SNPs, and compared with the DEG sliding window graphs, show the co-occurrence of DEG and SNP clustering (fig. 2*B*). No significant SNPs were found between resistant and sensitive plants for the HPPD comparisons. The reason for a lack of SNP clustering in the HPPD comparisons may be due to the more complex nature of this resistance trait, because it has been documented to be a multigenic trait in these populations (Murphy and Tranel 2019).

### ASE Analysis

**Fig. 3:**
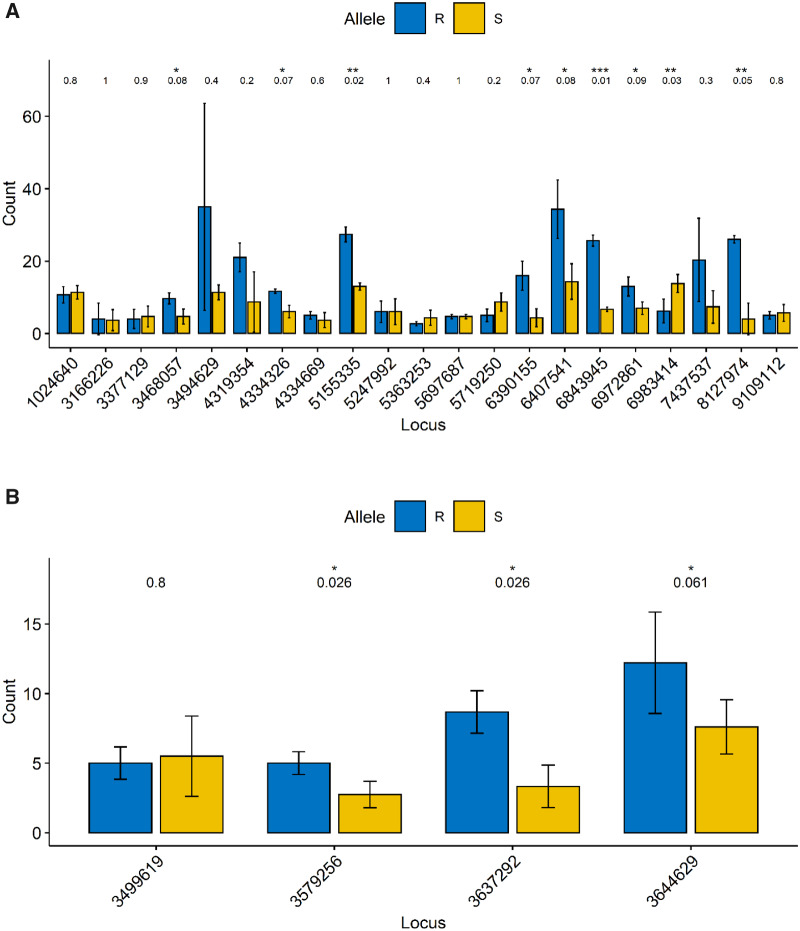
Allele-specific expression of all SNPs in the scaffold 4 hotspot region for (*A*) the NEB population and (*B*) the CHR population. The location of each SNP is given across the *x* axis and the results of a *t*-test for differential expression between the R and S allele (Benjamini and Hochberg adjusted *P*-value) is given above the bars for each locus.

The clustering of condition-specific SNPs with regions of differential gene expression suggested the occurrence of ASE. An ASE is defined as a form of allelic imbalance, wherein one parental allele is preferentially expressed over another allele (Knight 2004). In the scaffold 4 cluster, nine SNPs were found to be statistically significantly differentially expressed for NEB (fig. 3*A*). For all but one, the R allele had significantly higher expression than the S allele, perhaps indicating some *cis*-acting factor associated with this region, controlling expression. For the CHR population, there were four SNPs that occurred in this scaffold 4 region in heterozygous individuals and three showed significantly different expression between the two alleles (fig. 3*B*), again with the R allele showing higher expression than the S allele. ASE may also be occurring in other places along this region, but only the SNPs that were found to occur in a heterozygous state across three or more individuals were included in this analysis.

### *Cytochrome 81E8* Phylogenetic Analysis

**Fig. 4:**
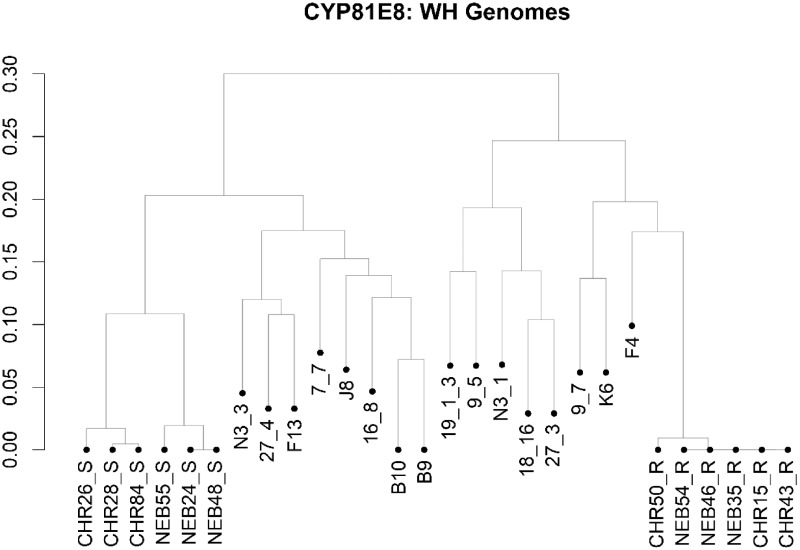
A phylogenetic tree of *cytochrome P450 81E8* in an arbitrary subset of *A. tuberculatus* populations from Illinois, Nebraska, Missouri, and Canada. Samples from this study are indicated with their population name (“CHR” or “NEB”) as well as their 2,4-D phenotypic response. Samples beginning with a number or “N3” originated from Ontario and samples beginning with “B,” “F,” “J,” or “K” originated from Illinois and Missouri.

Phylogenetic analysis of the CYP81E8 gene revealed the evolutionary relatedness of each *CYP81E8* allele from both the CHR and NEB populations and other *A. tuberculatus* populations from Illinois, Missouri, and Canada. As seen in figure 4, the *CYP81E8* alleles from CHR and NEB separated into three groups representing 1) the 2,4-D sensitive allele from NEB; 2) the 2,4-D sensitive allele from CHR; and 3) the 2,4-D resistant allele in both CHR and NEB. The separation of the wildtype sensitive alleles from CHR and NEB along with the tight clustering of the 2,4-D resistance-associated *CYP81E8* from CHR and NEB provides good evidence that the R allele in both populations has a common evolutionary origin.

## Discussion

### Herbicide Resistance Candidate Genes

Strong candidate genes for metabolic-based herbicide resistance were found for 2,4-D in both the CHR and NEB populations in this study. Both a cytochrome P450 (*CYP81E8*) and an ABC transporter (*ABCC10*) showed consistent overexpression in 2,4-D resistant plants compared with 2,4-D sensitive plants. These results support earlier work that found 2,4-D resistance in the NEB population was likely mediated by a cytochrome P450, because the cytochrome P450 inhibitor malathion reversed the resistance phenotype (Figueiredo et al. 2018). Follow-up work is needed to validate these genes in vitro, but the *CYP81E8* appears particularly promising. The putative resistance allele of this gene cosegregated with additional resistant plants from F_2_ populations (data not shown), and fine mapping is currently underway.

Our findings for HPPD-inhibitor resistance, however, were less clear. One candidate gene, a *UDP-glucose flavonoid 3-O-glucosyltransferase*, was confirmed to be overexpressed in tembotrione-resistant plants compared with tembotrione-sensitive plants. The primary functional annotation of this gene shows it to be involved in fruit ripening, but additional work has shown it to possibly participate in xenobiotic metabolism by glycosylation of exogenous substances (Griesser et al. 2008). The lack of additional candidate HPPD-inhibitor-resistance genes may be due to its multigenic nature (Oliveira et al. 2018), making it difficult to identify the resistance loci. Additionally, our RNA-seq approach focused primarily on identifying genes contributing to resistance via constitutive differential expression, potentially missing other resistance-conferring changes between the plants. A recent RNA-seq study looking into mesotrione resistance in *A. tuberculatus* did include treated plants and found some evidence of induced expression of *cytochrome P450a* in resistant plants, compared with sensitive plants (Kohlhase et al. 2019). However, the final list of DETs in this study was ∼4,800, making the identification of causative resistance genes difficult. Work using a genetic mapping approach to identify HPPD-inhibitor resistance genes in the NEB and CHR populations is currently underway.

Identification of coexpression networks was not extensively pursued in this work due to the fact that plants were not treated with herbicide prior to RNA-seq. Without this shared treatment, it is unlikely that coexpression analysis would yield anything meaningful, because it would measure the random expression differences across the two populations. Indeed, initial forays into coexpression networks yielded no informative results.

### Regulation of Herbicide Resistance

In addition to the identification of herbicide resistance gene candidates, this data also reveal some insights into the regulation of herbicide resistance. The physical clustering of DEGs observed for 2,4-D resistance provides evidence for coexpression of colocalized genes, a phenomenon that has been observed in many other species, including yeast (Cohen et al. 2000), *Arabidopsis* (Williams and Bowles 2004), *C. elegans* (Chen and Stein 2006), and human (Trinklein et al. 2003). Although several of these coexpression clustering examples are found between neighboring gene pairs, coexpression across longer chromosomal intervals has also been reported (Lercher and Hurst 2006; Reimegård et al. 2017). The ability of herbicides to reshape the genomic landscape of weedy species has been recently documented in *Ipomoea purpurea*, wherein evidence of selective sweeps was found in five genomic regions within glyphosate-resistant populations (Van Etten et al. 2020). Interestingly, enrichment for herbicide detoxification genes was apparent within these regions.

One major implication of this clustering is the likelihood of a shared mechanism of gene regulation for these regions. Regulation of gene expression is a complex process, involving the selective interaction of transcription factors with enhancers, the opening and closing of chromatin to allow/prevent transcription, and the interaction between these two processes (Voss and Hager 2014). In this study, we examined the upstream regions of all DEGs and looked for overrepresentation of TFBSs, but found no evidence of shared enhancer elements (data not shown). Previous work looking into regulation mechanisms for physically clustered, coexpressed genes has shown that coexpressed gene pairs are often regulated by shared transcription factors, whereas larger regions of shared expression across 10 − 20 genes are influenced by a change in the chromatin structure (Batada et al. 2007). However, only a few examples have been studied so far and the interdependent nature of regulatory mechanisms makes it difficult to ascertain direct causes of gene expression. It also should be noted that we cannot rule out that the observed physical clustering of co-expressed genes occurred as a result of linkage in the segregating F_2_ plants.

The differential expression of genes in scaffold 4 is associated with 2,4-D resistance in both *A. tuberculatus* populations in this study, but how many genes in this region actually contribute to the phenotype is unknown. A recent study of coexpression genes in humans (Kustatscher et al. 2017) found that despite high levels of coexpression between neighboring genes at the transcript level, only a small fraction (3%) had similar abundances at the protein level. Regardless of whether all differentially regulated genes in the scaffold 4 region contribute to resistance, the discovery of these expression clusters provides several benefits to transcriptomics researchers. As stated previously, they deliver insight into the regulatory mechanism responsible for the expression differences leading to resistance. They also provide an excellent forensic tool for narrowing down to the causal resistance gene. Finding a region of coexpression across individuals with a shared phenotype indicates some level of selection on that region, likely pointing to a gene within that region that is responsible for the phenotype.

## Conclusion

In summary, this study presents an intriguing story of nontarget-site herbicide resistance evolution in *A. tuberculatus*. The combination of RNA-seq and genomic data to unravel the patterns of gene expression in resistant plants has revealed colocalized, coexpression clusters for 2,4-D resistance in both populations. In at least one of these clusters, ASE is occurring, presenting a model in which *cis*-acting genetic variation is the cause of this differential expression. Future work building off this project has the potential to enhance our understanding of the regulatory systems underlying herbicide resistance. Additionally, for one of the genes found in this study (*CYP81E8*), a phylogenetic analysis revealed very close relatedness of the resistance-associated allele between the CHR and NEB populations despite no such relatedness found in the sensitive-associated allele, suggesting a common evolutionary origin despite the geographic separation of the two populations.

## Supplementary Material

Supplementary data are available at *Genome Biology and Evolution* online.

## Supplementary Material

evaa191_Supplementary_Data
