# Supplementary material for: Coexpression Clusters and Allele-Specific Expression in Metabolism-Based Herbicide Resistance
Source: Genome Biol Evol. 2020 Sep 11;12(12):2267–78. doi: 10.1093/gbe/evaa191 (PMC7738748; doi:10.1093/gbe/evaa191)
Supplement: evaa191_Supplementary_Data [file evaa191_supplementary_data.zip › Supplementary_Material.docx]

Supplementary Material

**slidingWindowPlots.R**

## Sliding window plot of DEG counts across all 16 chromosome-level scaffolds

# Import results from kallisto/sleuth differential expression analysis (e.g. DEGs found in NEB 2,4-D R vs S analysis)

DEG_NEB24D <- read.table("NEB24D_DEGs.txt", header = TRUE, sep = "\t")

DEG_NEB24D$SCAFFOLD = sapply(strsplit(as.character(DEG_NEB24D$scaffold),"\\_"),"[[",2) # Pull out scaffold number from ID field

# Add placeholders for scaffold if no DEG present on scaffold

y <- expand.grid(SCAFFOLD = 1:16)

DEG_NEB24D <- merge(DEG_NEB24D,y,all = TRUE)

# Add in scaffold lengths

DEG_NEB24D$length[DEG_NEB24D$SCAFFOLD == 1] = 38124660

DEG_NEB24D$length[DEG_NEB24D$SCAFFOLD == 2] = 35657244

DEG_NEB24D$length[DEG_NEB24D$SCAFFOLD == 3] = 30204323

DEG_NEB24D$length[DEG_NEB24D$SCAFFOLD == 4] = 28349311

DEG_NEB24D$length[DEG_NEB24D$SCAFFOLD == 5] = 25672467

DEG_NEB24D$length[DEG_NEB24D$SCAFFOLD == 6] = 24628041

DEG_NEB24D$length[DEG_NEB24D$SCAFFOLD == 7] = 24364990

DEG_NEB24D$length[DEG_NEB24D$SCAFFOLD == 8] = 23766980

DEG_NEB24D$length[DEG_NEB24D$SCAFFOLD == 9] = 22691259

DEG_NEB24D$length[DEG_NEB24D$SCAFFOLD == 10] = 22670516

DEG_NEB24D$length[DEG_NEB24D$SCAFFOLD == 11] = 22280117

DEG_NEB24D$length[DEG_NEB24D$SCAFFOLD == 12] = 22052327

DEG_NEB24D$length[DEG_NEB24D$SCAFFOLD == 13] = 20679869

DEG_NEB24D$length[DEG_NEB24D$SCAFFOLD == 14] = 20190685

DEG_NEB24D$length[DEG_NEB24D$SCAFFOLD == 15] = 17522127

DEG_NEB24D$length[DEG_NEB24D$SCAFFOLD == 16] = 16951160

### Scaffold plotting function (adapted from https://github.com/ctb/edda/blob/master/doc/tutorials-2012/files/plot_allele_freq_data.R)

plot.sliding.window.DEG <- function(scaff, step.size, window.size, data.file) {

#Extract only the DEGs that are on the scaffold called by the function

scaffold.data <- data.file[data.file$SCAFFOLD==scaff,]

#Set up the sliding windows and count up the number of DEGs in each window

positions.x <- seq(from=1, to=max(scaffold.data$length), by=step.size) #Vector of all positions along scaffold, from start to finish

DEG.counts <- rep(NA, length(positions.x)) #Empty vector to be filled with the DEG counts

for (cur.pos in 1:length(positions.x)) { #Loop over each position…

window.min.x <- positions.x[cur.pos] - (window.size / 2) #setting the window start position…

window.max.x <- positions.x[cur.pos] + (window.size / 2) #and stop position

scaffold.data1 <- scaffold.data[complete.cases(scaffold.data), ] #Exclude NA's

#Extract a subsetted data frame that has only the DEGs within our bin

cur.data <- scaffold.data1[(scaffold.data1$start >= window.min.x) & (scaffold.data1$start < window.max.x),]

#Count how many DEGs we found in this region and store it in the appropriate slot of DEG.counts

DEG.counts[cur.pos] <- count(cur.data)

}

#Set up the plot window

plot(x=NULL, y=NULL, xlim=c(1, max(positions.x)), ylim=c(0,30), xaxt='n')

#Add in x labels

axis(1, at=positions.x, labels=formatC(positions.x/1000000.0, format = "f", digits = 1), las=1, padj = -0.6)

#Plot the actual line

lines(x=positions.x, y=DEG.counts, col="red", lwd = 2)

#Add in scaffold number at top

mtext(scaff, side=3, line=-2, cex = 1.4)

}

#Output tiff image

tiff(file="NEB_24D_SWplot.tiff", width = 8, height = 8, units = "in", pointsize = 16, res = 600)

op <- par(mfrow = c(4,4), #Tell R to divide the plot window into 16 panels

oma = c(5,4,3,0) + 0.1,

mar = c(1,1,1,1) + 0.1)

for (i in 1:16) {

plot.sliding.window.DEG (scaff = i, step.size = 500000, window.size = 500000, data.file = DEG_NEB24D)

}

title(xlab = "Scaffold location (Mbp)", ylab = "Number of DEGs", outer = TRUE, line = 2, cex.lab = 1.5, main = "NEB 2,4-D DEGs")

par(op)

dev.off()

##########################################

## Sliding window plot of SNP counts across all 16 chromosome-level scaffolds

# Import results from PLINK association test (e.g. significant SNPs between NEB 2,4-D R vs S)

NEB24D = read.table('NEB_24D.assoc.mperm2.tab', header=TRUE, sep = "\t", row.names=NULL)

colnames(NEB24D) = c("IGNORE","SCAFFOLD","BP","PVAL","P")

SNP_NEB24D <- subset(NEB24D, NEB24D$P<=0.05) #Only plot significant SNPs (adj. Pvalue = 0.05)

# Add placeholders for scaffold if no SNP present on scaffold

y <- expand.grid(SCAFFOLD = 1:16)

SNP_NEB24D <- merge(SNP_NEB24D,y,all = TRUE)

# Add in scaffold lengths

SNP_NEB24D$length[SNP_NEB24D$SCAFFOLD == 1] = 38124660

SNP_NEB24D$length[SNP_NEB24D$SCAFFOLD == 2] = 35657244

SNP_NEB24D$length[SNP_NEB24D$SCAFFOLD == 3] = 30204323

SNP_NEB24D$length[SNP_NEB24D$SCAFFOLD == 4] = 28349311

SNP_NEB24D$length[SNP_NEB24D$SCAFFOLD == 5] = 25672467

SNP_NEB24D$length[SNP_NEB24D$SCAFFOLD == 6] = 24628041

SNP_NEB24D$length[SNP_NEB24D$SCAFFOLD == 7] = 24364990

SNP_NEB24D$length[SNP_NEB24D$SCAFFOLD == 8] = 23766980

SNP_NEB24D$length[SNP_NEB24D$SCAFFOLD == 9] = 22691259

SNP_NEB24D$length[SNP_NEB24D$SCAFFOLD == 10] = 22670516

SNP_NEB24D$length[SNP_NEB24D$SCAFFOLD == 11] = 22280117

SNP_NEB24D$length[SNP_NEB24D$SCAFFOLD == 12] = 22052327

SNP_NEB24D$length[SNP_NEB24D$SCAFFOLD == 13] = 20679869

SNP_NEB24D$length[SNP_NEB24D$SCAFFOLD == 14] = 20190685

SNP_NEB24D$length[SNP_NEB24D$SCAFFOLD == 15] = 17522127

SNP_NEB24D$length[SNP_NEB24D$SCAFFOLD == 16] = 16951160

### Scaffold plotting function (adapted from https://github.com/ctb/edda/blob/master/doc/tutorials-2012/files/plot_allele_freq_data.R)

plot.sliding.window.SNP <- function(scaff, step.size, window.size, data.file) {

#Extract only the SNPs that are on the scaffold called by the function

scaffold.data <- data.file[data.file$SCAFFOLD==scaff,]

#Set up the sliding windows and count up the number of SNPs in each window

positions.x <- seq(from=1, to=max(scaffold.data$length), by=step.size) #Vector of all positions along scaffold, from start to finish

SNP.counts <- rep(NA, length(positions.x)) #Empty vector to be filled in with SNP counts

for (cur.pos in 1:length(positions.x)) { #Loop over each position...

window.min.x <- positions.x[cur.pos] - (window.size / 2) #...setting the window start position...

window.max.x <- positions.x[cur.pos] + (window.size / 2) #...and stop position

scaffold.data1 <- scaffold.data[complete.cases(scaffold.data), ] #Exclude NA's

#Extract a subsetted data frame that has only the SNPs within our bin

cur.data <- scaffold.data1[(scaffold.data1$BP >= window.min.x) & (scaffold.data1$BP < window.max.x),]

#Count how many SNPs we found in this region and store it in the appropriate slot of SNP.counts

SNP.counts[cur.pos] <- count(cur.data)

}

#Set up the plot window

plot(x=NULL, y=NULL, xlim=c(1, max(positions.x)), ylim=c(0,50), xaxt='n')

#Add in x labels

axis(1, at=positions.x, labels=formatC(positions.x/1000000.0, format = "f", digits = 1), las=1, padj = -0.6)

#Plot the actual line

lines(x=positions.x, y=SNP.counts, col="red", lwd = 2)

#Add in scaffold number at top

mtext(scaff, side=3, line=-2, cex = 1.4)

}

#Output tiff image

tiff(file="NEB_24D_SWplot_SNP.tiff", width = 8, height = 8, units = "in", pointsize = 16, res = 600)

op <- par(mfrow = c(4,4), #Tell R to divide the plot window into 16 panels

oma = c(5,4,3,0) + 0.1,

mar = c(1,1,1,1) + 0.1)

for (i in 1:16) {

plot.sliding.window.SNP(scaff = i, step.size = 500000, window.size = 500000, data.file = SNP_NEB24D)

}

title(xlab = "Scaffold location (Mbp)", ylab = "Number of SNPs", outer = TRUE, line = 2, cex.lab = 1.5, main = "NEB 2,4-D SNPs")

par(op)

dev.off()

Supplementary Figure 1: Photos of representative plants for visual rating scale for both tembotrione (top) and 2,4-D (bottom). Plants receiving a visual rating of 1, 2, 3, 4, and 10 are shown.


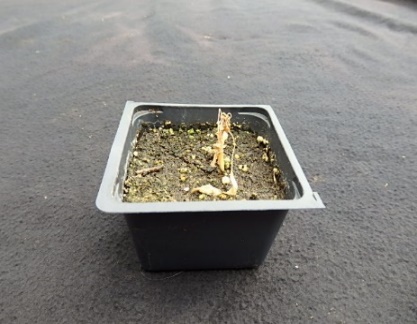

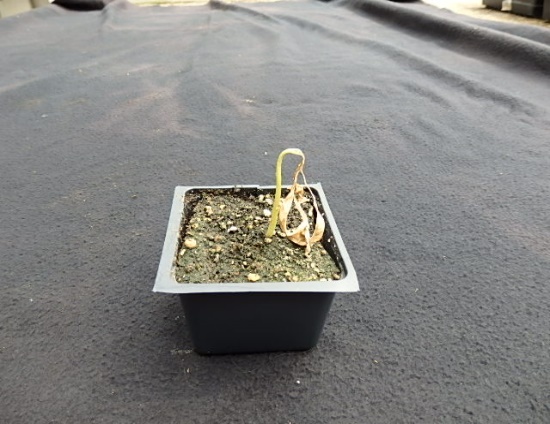

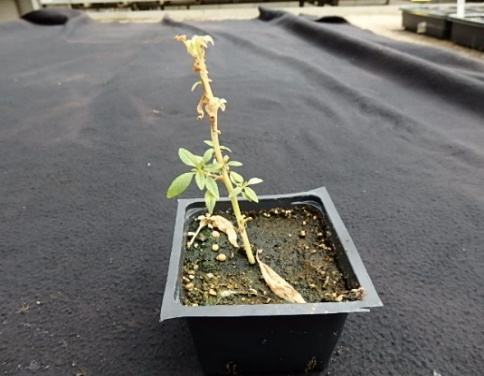

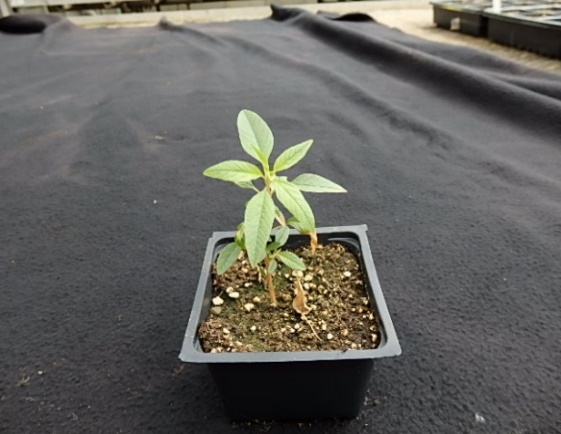

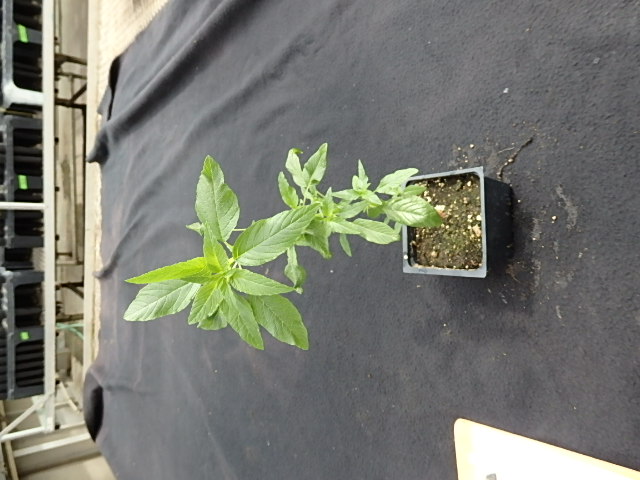


**1**

**2**

**3**

**4**

**10**


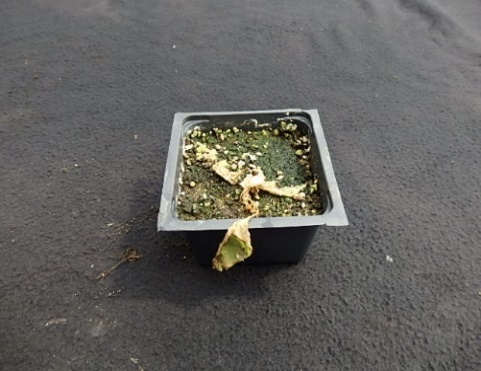

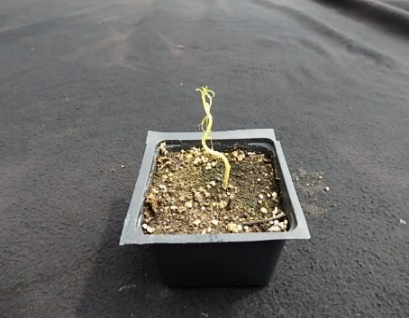

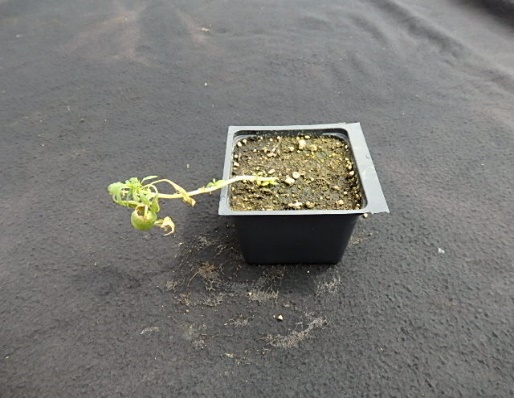

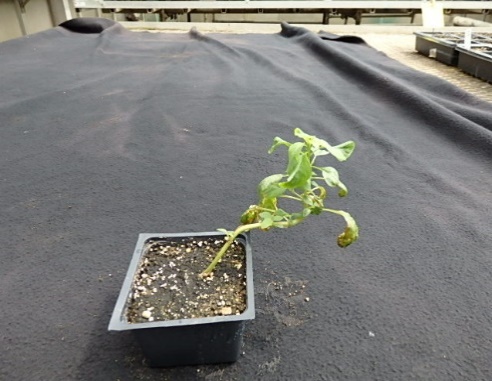

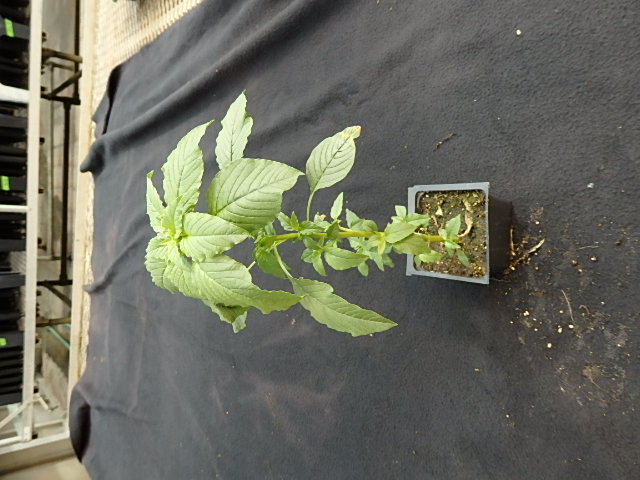


**1**

**2**

**3**

**4**

**10**

**Tembotrione**

**2,4-D**
